# Hybrid halide perovskite neutron detectors

**DOI:** 10.1038/s41598-021-95586-3

**Published:** 2021-08-30

**Authors:** Pavao Andričević, Gábor Náfrádi, Márton Kollár, Bálint Náfrádi, Steven Lilley, Christy Kinane, Pavel Frajtag, Andrzej Sienkiewicz, Andreas Pautz, Endre Horváth, László Forró

**Affiliations:** 1 Laboratory of Physics of Complex Matter, Ecole Polytechnique Fédérale de Lausanne (EPFL), 1015 Lausanne, Switzerland; 2 ISIS Pulsed Neutron and Muon Source, STFC Rutherford Appleton Laboratory, Harwell Oxford, Didcot, OX11 0QX UK; 3 Laboratory of Reactor Physics and Systems Behaviour, Ecole Polytechnique Fédérale de Lausanne (EPFL), 1015 Lausanne, Switzerland; 4 ADSresonances Sàrl, 1028 Préverenges, Switzerland; 5 Nuclear Energy and Safety, Paul Scherrer Institute, 5232 Villigen PSI, Switzerland

**Keywords:** Experimental nuclear physics, Electronic properties and materials, Solar cells, Electronic devices, Sensors and biosensors

## Abstract

Interest in fast and easy detection of high-energy radiation (x-, γ-rays and neutrons) is closely related to numerous practical applications ranging from biomedicine and industry to homeland security issues. In this regard, crystals of hybrid halide perovskite have proven to be excellent detectors of x- and γ-rays, offering exceptionally high sensitivities in parallel to the ease of design and handling. Here, we demonstrate that by assembling a methylammonium lead tri-bromide perovskite single crystal (CH_3_NH_3_PbBr_3_ SC) with a Gadolinium (Gd) foil, one can very efficiently detect a flux of thermal neutrons. The neutrons absorbed by the Gd foil turn into γ-rays, which photo-generate charge carriers in the CH_3_NH_3_PbBr_3_ SC. The induced photo-carriers contribute to the electric current, which can easily be measured, providing information on the radiation intensity of thermal neutrons. The dependence on the beam size, bias voltage and the converting distance is investigated. To ensure stable and efficient charge extraction, the perovskite SCs were equipped with carbon electrodes. Furthermore, other types of conversion layers were also tested, including borated polyethylene sheets as well as Gd grains and Gd_2_O_3_ pellets directly engulfed into the SCs. Monte Carlo N-Particle (MCNP) radiation transport code calculations quantitatively confirmed the detection mechanism herein proposed.

## Introduction

Over the last decade, hybrid organic–inorganic halide perovskites, CH_3_NH_3_PbX_3_ (X = I, Br, Cl), have not only revolutionized the field of emerging photovoltaic technologies but also demonstrated their great potential for other optoelectronic applications^1^, including, photodetectors^2–4^, light emitting devices^5,6^, gas sensors^7^, in data storage^8^, thermo-electrics^9^, and sensitive radiation detectors^10–19^. In the latter application, the hybrid perovskite CH_3_NH_3_PbBr_3_ (MAPbBr_3_) seems to be gaining particular popularity as it meets a significant portion of the requirements for a semiconducting ionizing radiation detector. In particular, the chemical formula of MAPbBr_3_ contains elements of large atomic weight (high-Z), such as Pb and Br, thus leading to a high material density (~ 4 g cm^−3^), which is necessary for efficient impact ionization. Moreover, it has been shown that high-quality large MAPbBr_3_ crystals, weighting even more than one kilogram, can be grown^20^. Such crystals are characterized by a unique correlation of relatively low trap concentration and high resistivity. They also reveal high mobility-lifetime products (*µτ*)^17^, which is a prerequisite for efficient charge collection. The radiation detection is performed by simple resistivity measurement. Combined with their low cost and easy fabrication this makes them very interesting for detector applications.

Numerous literature reports documented alpha-^21^, beta-particles^22^, X-ray^10,12–15,23–27^ and γ-ray^11,16–20,28–31^ detection by perovskite-based devices. All of them revealed exceptional performance, being capable of detecting a wide range of radiation energies and dose-rates, with sensitivities often equal or even better than the *best-in-class* semiconducting materials^32^.

It is worth mentioning, however, that one important radiation is not included in the above list, *i.e.,* neutron radiation. It is widely accepted that sensitive detection of this type of radiation is of great importance for the technology of nuclear reactors. In addition, beyond detection purposes, neutron-sensitive capturing devices could also serve for energy harvesting^31^.

This last-mentioned issue is important because in typical nuclear reactors, apart from the high flux broad spectrum gamma radiation, there is also the thermal neutron flux of 10^12^–10^14^ n/cm^2^/s^33^, which is also accompanied by the leaking neutron flux of ~ 10^10^–10^11^ n/cm^2^/s^34^. From the point of view of the nuclear chain reaction, these leaking neutrons are wasted. Moreover, they can also cause unwanted activation and structural degradation of the reactor components.

In this context, pertinent questions arise: (1) Could perovskite-based devices detect these leaked neutrons? (2) Could one use them for energy production? (3) What would be the effect of the neutrons on the perovskite radiation detectors in the reactor? We address these issues in the current work.

**Figure 1:**
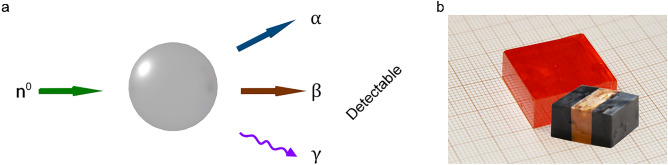
(**a**) Schematic representation of the principle of neutron radiation conversion into various forms of secondary radiation that can be more easily detected. (**b**) Photo showing two MAPbBr_3_ SCs of relatively large volumes (~ 2 × 2 × 1 cm^3^), one of which is equipped with graphite spray electrodes to work as a radiation detector.

Generally, neutron detection requires a conversion material to convert the charge-less neutrons into charged particles or γ-ray photons (Fig. 1a) that can subsequently be collected at the device terminals (electrodes). Typically, to detect neutrons, Geiger-Muller type detectors filled with conversion gaseous media, such as ^3^He or BF_3_, are used. Recently, to detect neutrons, aerosol-filled detectors or lining with typically isotopes of ^10^B, ^6^Li or enriched ^235^U have been developed^35^. Gas-filled detectors operate in high voltage regimes, usually ranging from several hundred volts to several kilo-volts. Alternatively, in scintillator neutron detectors, the absorbed neutrons create charged particles in the scintillating material, which, in turn, excite the scintillating medium via one or more steps towards emitting visible light. Scintillation of MAPbBr_3_ crystals was recently reported only during X-ray irradiation at low temperatures^36^.

Semiconductor-based neutron detectors with lining or coating with neutron converter are also used. In this case, the charged particle emitted from the neutron absorber enters to the semiconductor device where further electron–hole pairs are generated and collected at the electrodes^37,38^. Doping these detectors with elements that could increase the neutron capture efficiency, for example ^10^B enriched hBN neutron detectors^39^, should be the way for increasing the detection efficiency of semiconductor-based devices.

**Figure 2:**
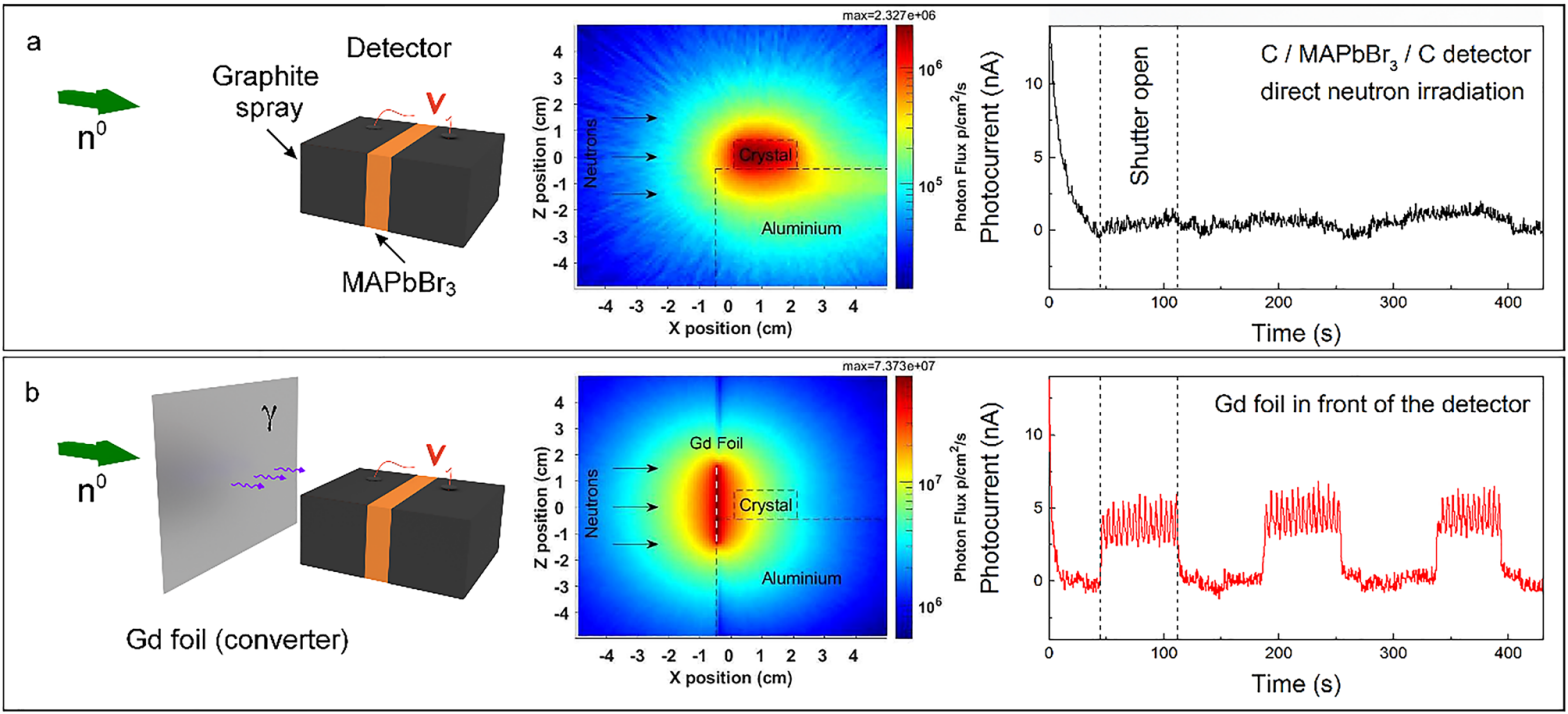
(**a**) Left panel: Schematic representation of the direct detection of neutron radiation by a MAPbBr_3_ SC equipped with graphite spray electrodes. Neutrons are converted to secondary radiation in the MAPbBr_3_ SC. Middle panel: The calculated photon flux map around the MAPbBr_3_ SCs positioned on an aluminium stage. Right panel: The photocurrent acquired as a function of time. The first three periods of shutter opening are shown. (**b**) Left panel: Schematic representation of the successful detection of neutron radiation by an assembly consisting of the Gd foil and the MAPbBr_3_ SC equipped with graphite spray electrodes. The Gd foil converts neutrons to detectable gamma rays. Middle panel: The calculated photon flux map around the MAPbBr_3_ SCs in the presence of the Gd foil (white dashes). Right panel: The photocurrent acquired as a function of time for the first three periods of shutter opening. Measurements were performed under ambient conditions, using 1 V pulsed bias voltage (200 ms between voltage pulses). The current peaks during the open shutter stages are the result of the 10 Hz repetition rate of the neutron beam sampled with the Keithley’s readout frequency.

In this work, single crystals (SCs) of methylammonium lead tri-bromide MAPbBr_3_ (MAPbBr_3_ SCs), were used to explore the detection of thermal neutrons (Fig. 1b). The first approach was based on the direct detection by irradiating the unaltered MAPbBr_3_ SCs with the neutron beam (Fig. 2a). In this case, the neutrons are converted to secondary radiation directly by the MAPbBr_3_ crystal due to nuclear interactions, for example via the prompt gamma production (n,γ), which ultimately generates charge carriers. In the second approach, a converter layer was positioned in front of the MAPbBr_3_ crystal inside the neutron beam to convert the neutrons to prompt gamma radiation (Fig. 2b). These γ-rays were then absorbed by the perovskite crystal, thus producing photo-generated charge carriers and leading to an increase in the device current. Other converter layers were also tested, from borated polyethylene sheets to Gd and Gd_2_O_3_ pallets, which were engulfed into the MAPbBr_3_ single crystal itself. In addition, SCs of methylammonium lead tri-iodide and -chloride (MAPbX_3_, X: I, Cl SCs) were also tested. While, similarly to its bromine counterpart, the MAPbI_3_ SC—based device demonstrated accurate and stable detection of the neutron beam, the detector based on the MAPbCl_3_ SC did not show sufficient sensitivity. Furthermore, the long-term operational stability, *i.e.,* radiation damage of the perovskite material, was tested under neutron irradiation. Since the neutron detectors, based on high-quality MAPbBr_3_ SCs manufactured by us, showed the best properties^20^, all the results presented hereinafter will concern the devices designed around MAPbBr_3_ SCs equipped with graphite spray (Fig. 1b) or carbon epoxy electrodes.

## Experimental section

The MAPbBr_3_ SCs used in the fabrication of the detector devices were grown using the inverse temperature crystallization method^20^. The SCs of a volume of 2 × 2 × 1 cm^3^ were coated by graphite spray on two sides leaving a small gap in-between (6 mm). Subsequently, these two graphite electrodes were contacted by carbon epoxy to copper wires, utilized as electrical leads.

To test the ability of neutron detection by the perovskite, the fabricated detectors were positioned in a direct beam of cold-thermal neutrons from 0.9 Å or ~ 101 meV to 15 Å or ~ 0.36 meV at the Polref reflectometer^40^ of the ISIS Neutron and Muon Source Facility. The instrument was used in a non-polarized mode, yielding a neutron flux of ~ 10^7^ n/cm^2^/s at the sample position. The Polref reflectometer was positioned at Target Station 2, thus neutron packages arrived at 10 Hz pulse frequency, after being moderated in a coupled grooved cold H_2_/CH_4_ moderator cell.

The detectors were biased in the 0–10 V range (Keithley 2400 source meter), depending on the crystal size, while measuring the current through the device. Care was taken to keep the crystal in darkness during the whole measurements to avoid the impact of visible light (in supporting information (SI) Fig. S1). The bias was pulsed every 200 ms, to achieve a stable current baseline, due to the tendency of perovskite material to undergo ion migration under constant voltage^41^, which would result in an increase of the device current with time also known as the polarization effect common in other radiation detector materials^18^.

## Results and discussion

When the MAPbBr_3_ SC-based device is exposed directly to the cold-thermal neutron beam the neutron capture reaction leaves nuclei in an unstable excited state. The excited nuclei relax through the emission of charged particles (*e.g*., α or β-particles) and gamma rays (Fig. 1a). The generated gamma photons can interact with the crystal material, depositing a part of their energy, thus leading to the subsequent formation of charge carriers via electron–hole pair-production, Compton scattering or photoelectric effect. Our Monte Carlo calculations for the elements in the SC identified two major pathways: 92.1% of the total deposited energy is resulting from photons and ultimately electrons and 7.29% from neutrons. Figure 2a shows the calculated spatial distribution of the γ-photon flux inside and around the MAPbBr_3_ SC device. The highly energetic electrons on their few millimetres path in the crystal create electron–hole pairs, which contribute to the photocurrent. Calculations give that the 10^7^ n/cm^2^/s neutron flux is converted into 2 × 10^6^ γ/cm^2^/s photon flux in the MAPbBr_3_ SC device. This remarkable n → γ conversion efficiency, however, in the measurement resulted in a moderate device-current of about 1 nA upon opening the neutron beam shutter (Fig. 2a). Although, the gamma radiation induced by Bromine has many high-energy (6–8 MeV) photons (Fig. S2), but they are not very efficient in photocurrent generation because they easily escape the active detector volume. In general, low energy gamma photons are more beneficial in photoelectron generation, which in our experiment is linked to the neutron flux.

To boost the low-energy gamma radiation and the overall detector efficiency a converter layer was positioned in the neutron-beam in front of the perovskite SC. Neutrons from the beam interact with the layer, generating Gamma rays, which are in turn detected by the perovskite SC. Several known isotopes have large neutron absorption cross-sections like ^10^B, ^113^Cd, ^135^Xe, ^155^Gd, ^157^Gd, etc. For the first converter layer a 5 × 5 cm^2^ gadolinium (Gd) foil was chosen with a thickness of 0.25 mm (Fig. 2b). We used natural gadolinium (i.e. without isotope enrichment) because the ^155^Gd and ^157^Gd isotopes appear with 14.8% and 15.65% natural abundancies thus the foil offers a good balance between price and conversion efficiency. The neutron capture cross-sections of the two Gd isotopes are 60900 b and 254000 b respectively^42^. This extremely high cross-section comes with a wide range of gamma lines at relatively low energies (Fig. S2). The released energy or Q-values during the neutron capture in the two Gd isotopes are rather high 8.536 MeV and 7.937 MeV respectively and about 99% of the energy is radiated out in the form of photons^43^. The resulting gamma-flux is about 7 × 10^7^ p/cm^2^/s in the converter and exceeds 4 × 10^6^ p/cm^2^/s at the sample position. Moreover, these low energy gammas have similar energies as X-rays in already documented experiments with perovskite SC^10^, so their efficient absorption is expected. As seen in Fig. 2b, 5 nA photocurrents are measured for the MAPbBr_3_ sample under 1 V bias voltage. The converter should be thin to minimize the self-absorption of the gamma rays^44^. The 0.25 mm thin Gd foil used in our experiments shows some self-absorption revealed by the asymmetric gamma flux at two sides of the foil (Fig. S3). Applying a converter layer also increased the high energy electron flux in the crystals. Without the converter, the calculated electron flux was about 1 × 10^5^ e/cm^2^/s while with the Gd converter it was calculated to be around 1 × 10^6^ e/cm^2^/s. The calculated photon, electron and neutron flux distributions for all detectors as well as converters used in this work can be found in the SI (Figs. S4–S11).

**Figure 3:**
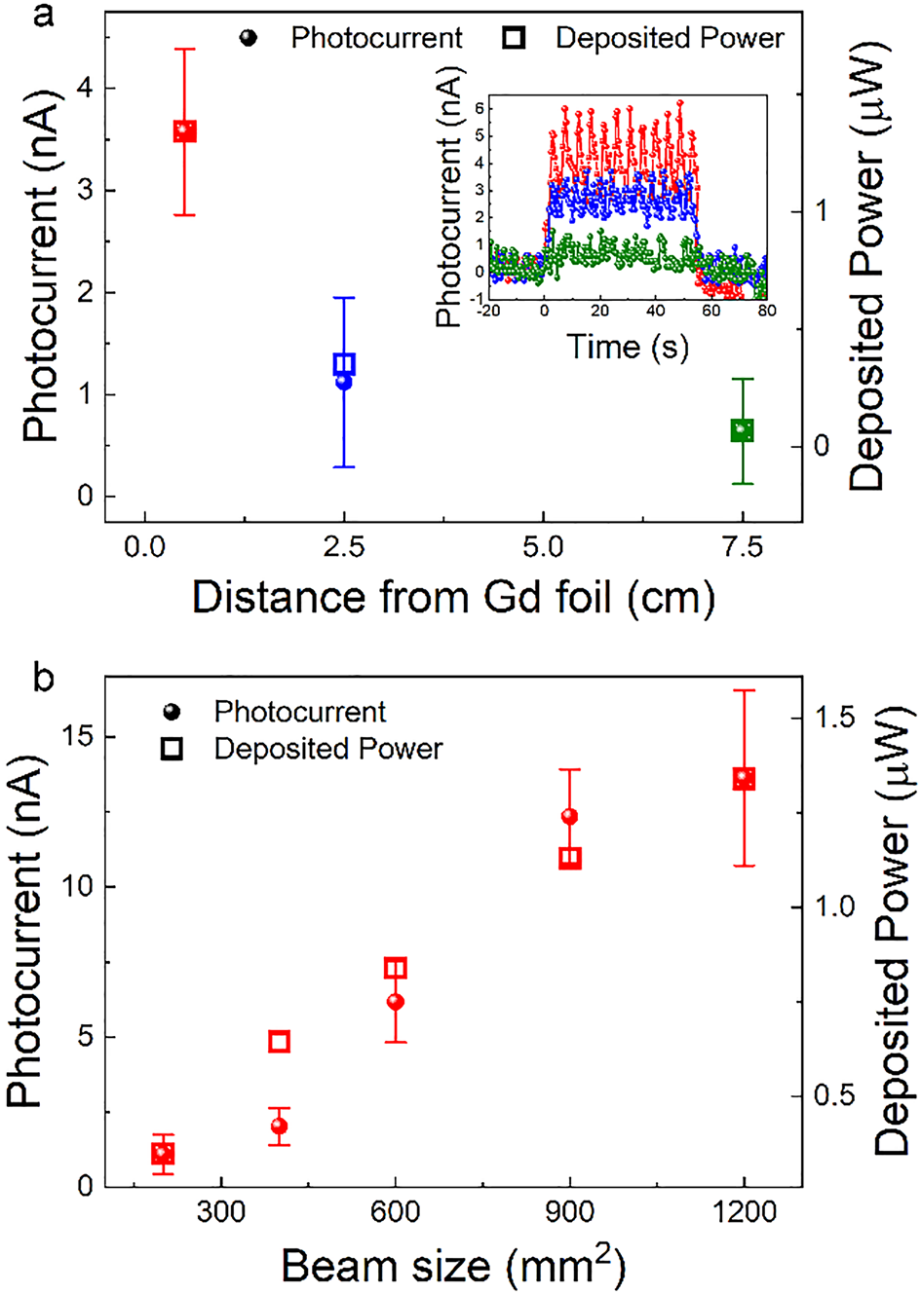
The dependence of the detected photo-current and the deposited power on: (**a**) the distance between the MAPbBr_3_ SC and the Gd foil and (**b**) the neutron beam size. Measurements were performed under ambient conditions, using 1 V pulsed bias voltage (200 ms between pulses). Inset in (**a**) shows the measured photocurrent while opening and closing the neutron beam shutter.

To provide further insight into the mechanism of neutron detection, the photocurrent was measured while changing both the distance of the SC from the foil as well as the beam (slit) size as shown in Fig. 3a, b, respectively. Since the Gd foil is acting as a radiation source, the perovskite SC was positioned as close to it as possible (0.5 cm) to achieve the highest dose rate of gamma irradiation. In addition, for similar reasoning, the slit size of the beam shutter was kept at its maximum (40 × 30 mm^2^). By changing these parameters, the variation of the observed photocurrent is in great agreement with our Monte Carlo calculations confirming the proposed detection mechanism (Fig. 3). In addition, due to the fact that one can correlate the beam size to different intensities of the neutron beam (knowing its flux). A linear dependence of the photocurrent with the neutron flux is estimated from Fig. 3b as shown in Fig. S12.

**Figure 4:**
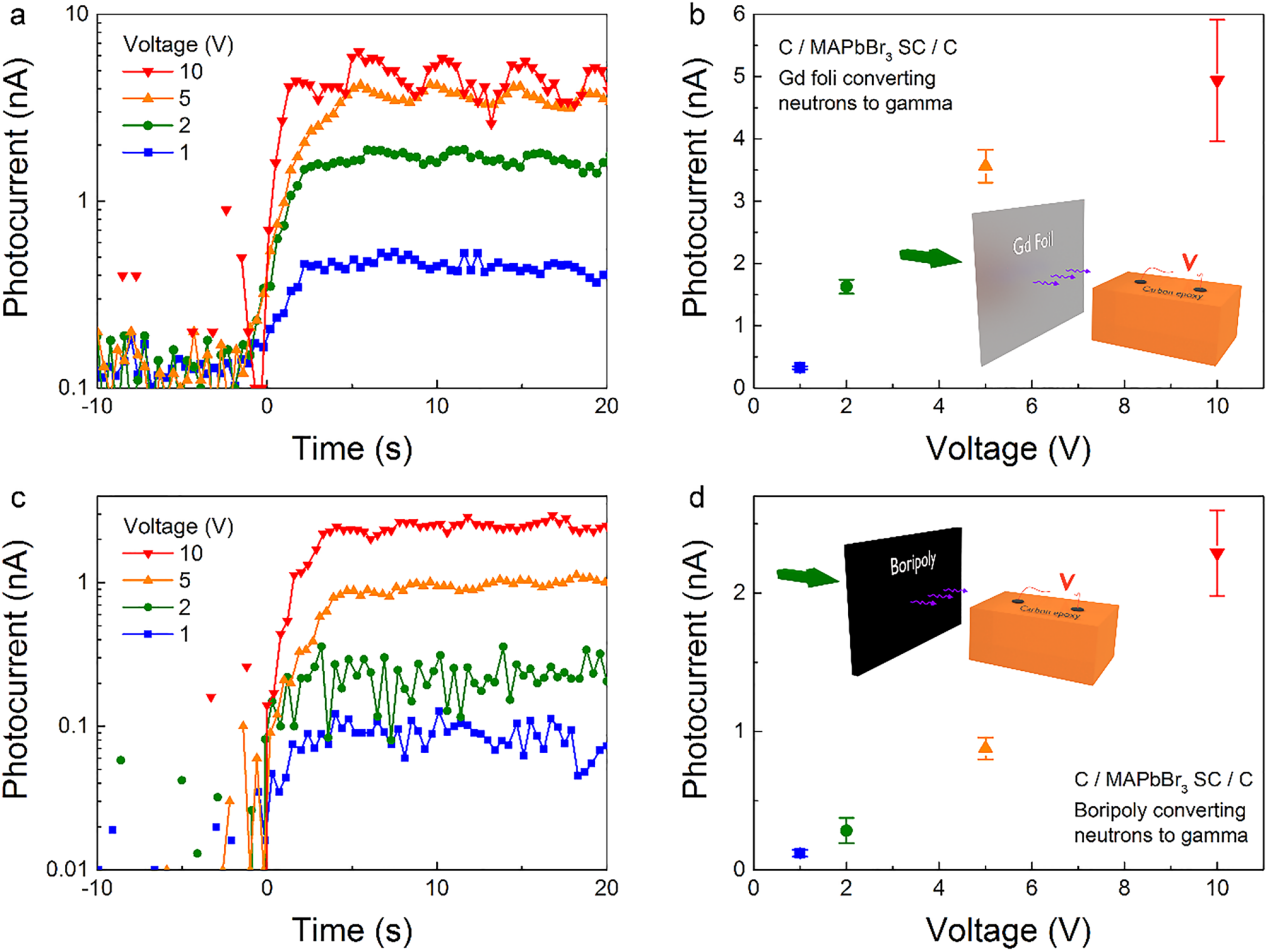
(**a**) Zoom in on the leading edge of the signal and (**b**) its dependence with various bias voltage for the Gd-foil/MAPbBr_3_ detector. Distance of crystal to foil is 2.5 cm. (**c**) Zoom in on the leading edge of the signal and (**d**) its dependence with various bias voltage for the Boripoly/MAPbBr_3_ detector. Distance of crystal to foil is 0.5 cm. Inset: Setup illustration.

Although the graphite spray electrode design offers many advantages for efficient charge collection, its capacitor-like architecture can induce ion migration inside the crystal^32^. This results in a continuous increase in current at higher bias voltages (> 1 V), cumulating in a decreased device performance. Therefore, alternatively, detectors were also fabricated from perovskite SCs with simple carbon epoxy electrodes. The Gd-foil/MAPbBr_3_ device exhibited a clear photocurrent response when opening the shutter as seen in Fig. 4a. Bias voltages from 1 up to 10 V were applied, resulting in an increase of photocurrent by an order of magnitude (Fig. 4b), even increasing the photocurrent value compared to the graphite spray electrode device (5 ×). However, at higher voltages the noise in the signal increases as well as the baseline drifts starts to be present. With a better electronic design, for example, asymmetric electrode materials resulting in a diode-like behaviour, these effects could be saturated, enabling higher voltages to be applied. MAPbI_3_ and MAPbCl_3_ SCs were also tested with the same electrode design and utilization of the Gd-foil converter. The iodide version was able to detect the beam, however, with much lower photocurrents (Fig. S13). This is most likely due to the smaller thickness (0.3 cm) that is, to the smaller active volume of the MAPbI_3_ crystals. Therefore, the better low energy deposition capability of MAPbI_3_ (Fig. S14) could not fully compensate for the smaller active volume. Furthermore, as seen in supporting Fig. S13, the wave effect of the current when the shutter is open (beam “on”) is much more enhanced in the case of MAPbI_3_. On the other hand, for the chloride sample, the photocurrent signal was in the range of the noise making the beam undetectable. This is interesting since a similar sized (2 cm × 2 cm × 1 cm) MAPbCl_3_ SC was used as for the bromine counterpart and the simulations of chlorine-containing crystal showed the highest direct conversion capability of neutrons to photons and electrons energy deposition (see supporting Table S1). This goes to show that for the overall device performance, nuclear behaviour is just one factor to take into account and that the crystal´s electrical properties are also important.

Besides gadolinium, ^10^B isotope has a high cross-section for the (n,α) reaction, which is followed by a gamma photon emission (~ 477 keV with 716 b)^45^. Therefore, a detector was fabricated replacing the Gd foil and inserting a 0.5 × 10 × 5 cm^3^ borated polyethylene (mixtures of B_4_C and polyethylene) sheet. The calculated gamma emission is depicted in supporting Fig. S4. As seen, it provides intermediate values of gamma-flux between the MAPbBr_3_ self-conversion and Gd foil converter. Indeed, a clear detection of the beam was achieved with this device, as well (Fig. 4c). As in the case for the Gd foil, photocurrent responses were measured at voltages from 1 to 10 V as seen in Fig. 4c. However, about a factor two lower overall values were attained (Fig. 4d). Lower photocurrent values can be partially explained by the lower gamma flux of the converter. Nevertheless, the gamma-flux dropped by an order of magnitude while the photocurrent only by a factor of two. This is explained by the higher efficiency of transforming the ^10^B gammas to photo-electrons which have energy practically only below about 477 keV (Figs. S14–S16).

**Figure 5:**
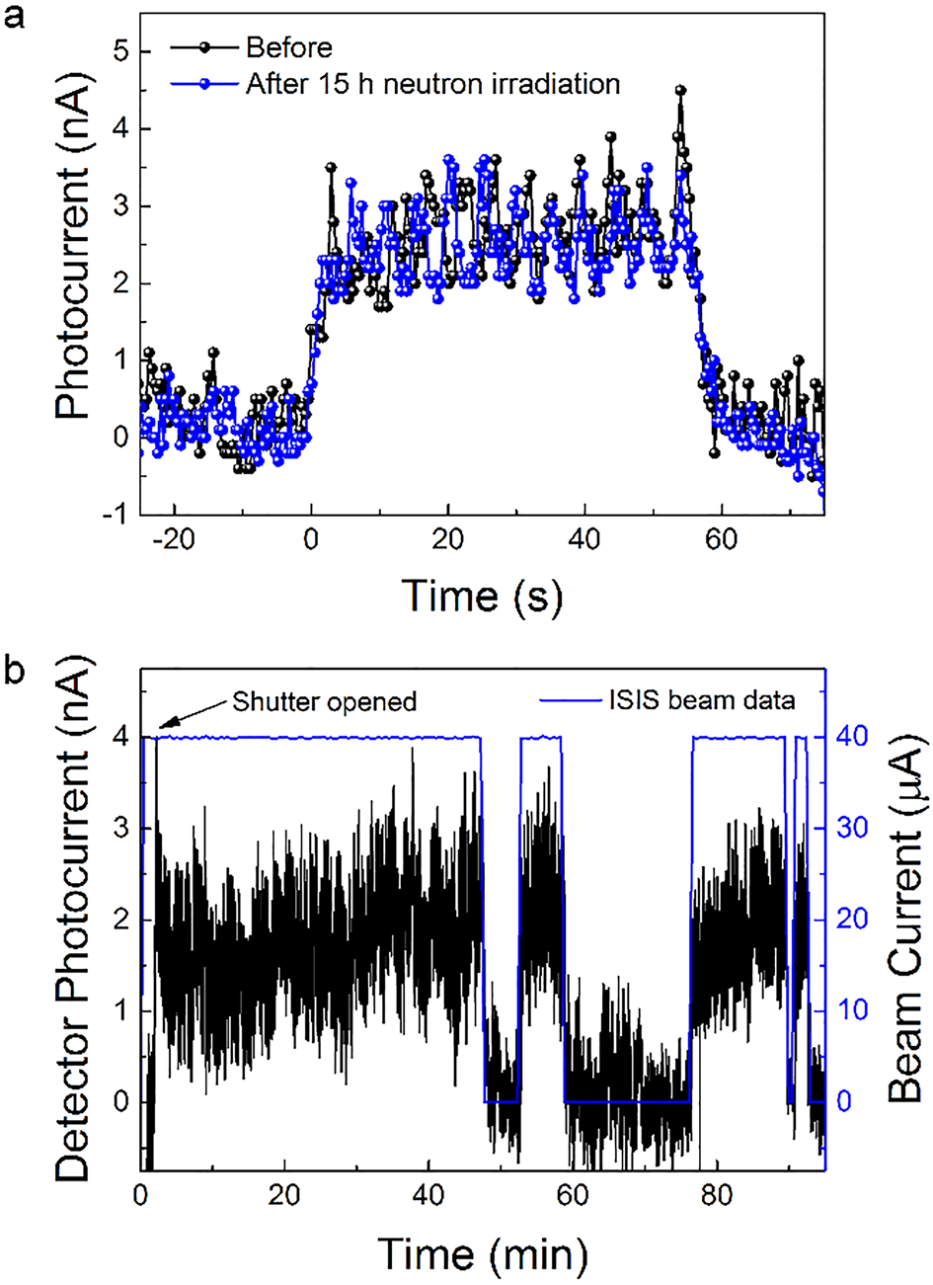
(**a**) Photocurrent response of the Gd-foil/MAPbBr_3_ SC detector to the neutron beam, before and after 15-h direct neutron irradiation of the perovskite SC. (**b**) Zoom in on a 15-h neutron irradiation of the Gd-foil/MAPbBr_3_ detector during “On-Offs” of the beam.

One important parameter of perovskite-based optoelectronic devices is their long-term stability. The instability of the material to humidity, high temperatures and light is still limiting these devices from full commercialization, especially perovskite solar cells. However, for radiation detection, all these environmental conditions can be easily overcome, as the devices can be encapsulated and kept in darkness, without screening the incoming radiation. On the other hand, radiation damage has to be taken into account. Both gamma-rays and neutrons can lead to displacement damage in all solid-state detectors^46^. In our previous work, MAPbBr_3_ devices were shown to work in operational conditions for more than 100 h under gamma irradiation, accumulating more than 200 Gy without any sign of degradation^32^. This excellent radiation tolerance was attributed to the ability of perovskite to self-heal these initiated defects on the nanoscale^47–49^. Nevertheless, perovskite solar cells were recently irradiated by fast neutrons, exhibiting permanent defects likely originating from severe atomic displacement^50^. However, the effect of low-energy neutrons, which is our focus, is still unexplored. Therefore, to test if the self-healing mechanism will still take place in the case of thermal neutrons, i.e. if we would see a decrease in device performance, a SC MAPbBr_3_ detector was irradiated for 15 h under the direct neutron beam. The deposited power estimated by MCNP is 0.224 µW, therefore the total absorbed energy during the neutron irradiation is 0.0121 J. As mentioned above 7.29% of this energy is deposited directly by neutrons through various nuclear processes and 92.1% is deposited by secondary photons and ultimately electrons. For comparison, when the Gd or the borated polyethylene converters were used, practically no energy was deposited by neutrons in the crystal. From the Gd foil to the crystal 93.8% of the deposited energy is transported by photons and only 6.2% by electrons. In the case of borated polyethylene this ratio is 99.8% and 0.2%, respectively. After the irradiation, there was no visible degradation of the crystal, and in addition the device detecting capabilities remained the same, as illustrated by Fig. 5a.

The long-term measurement was then repeated after inserting the Gd-foil converter in front of the MAPbBr_3_ SC, to test the operational stability of our neutron beam detector. A pulsing bias voltage of 1 V was applied (1 s every min) to reduce the baseline current variation with time. A clear “turn-on” and “off” current response is visible (Fig. S17) with as well some drops of current during the 15-h measurement. These drops, shown in a zoom-in of the long-term measurement in Fig. 5b, correspond to the loss of the neutron beam at the ISIS facility. As the current of the beam goes down, we have a decrease in the current through the detector. This great alignment is proof that our fabricated device can work as a neutron beam tracker. Nevertheless, a shift in the baseline is visible. This is not due to the degradation of the detector as was proven before, but to the electronics and bias voltage scheme. Improving the electronics could suppress the shift of the baseline, as well as suppress the “dark” current in general.

## Conclusion and outlook

Neutron detectors were fabricated using MAPbBr_3_ SCs as the sensing material. The high-sensitivity device architecture consisted of a converter layer, a gadolinium foil or a borated polyethylene sheet, which converted the incoming thermal neutrons to prompt gamma radiation. These gamma rays were then detected by the highly sensitive perovskite single crystals equipped with graphite spray electrodes. The detectors were able to work at low bias voltages and showed great stability, while working in operational conditions under irradiation for more than 15 h without any sign of degradation or device performance deterioration. The simplicity of the detection (by photocurrent measurement), low-cost fabrication process and stability under radiation makes these methylammonium lead tri-halide perovskites a good candidate for the next generation of neutron detectors.

**Figure 6:**
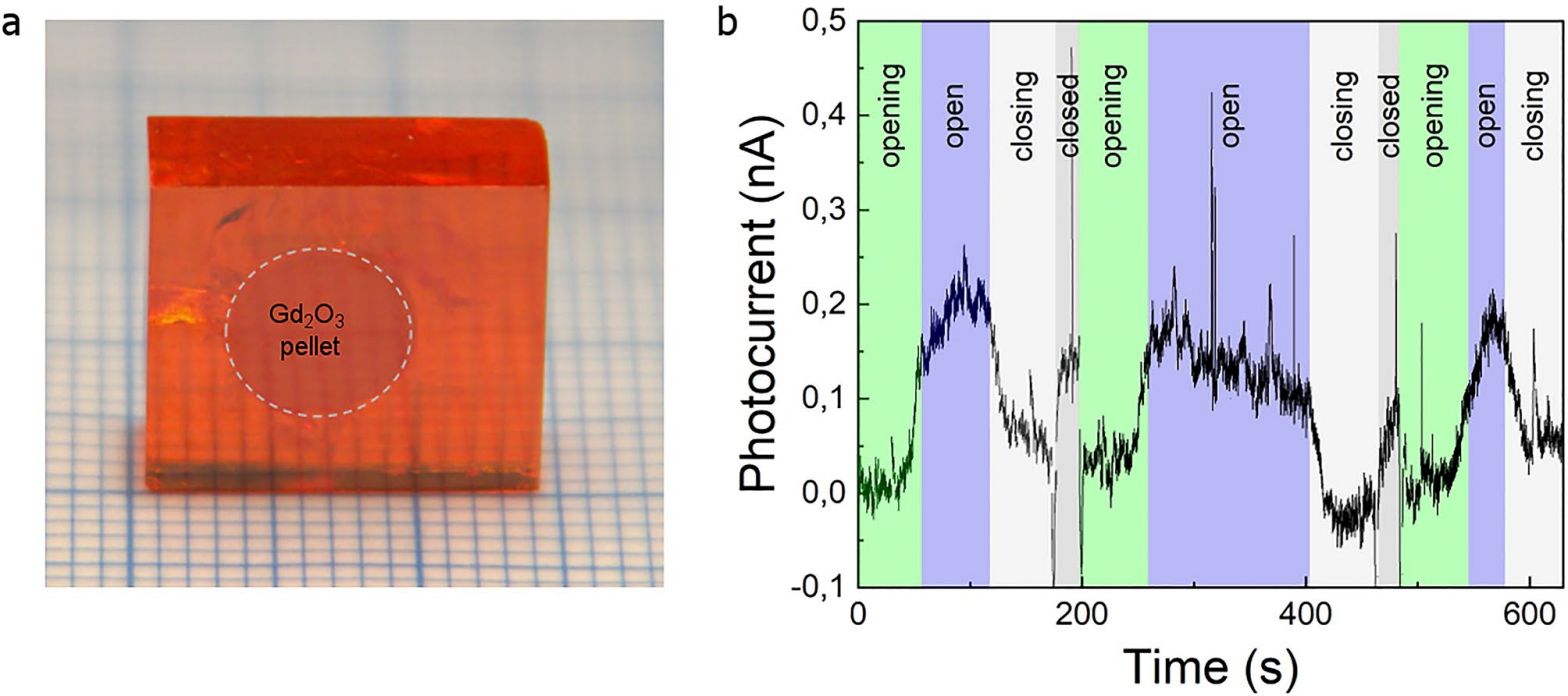
(**a**) Photo of a MAPbBr_3_ SC with a 190 mg Gd_2_O_3_ pressed pellet engulfed during the growth (for better visibility the contour of the pellet is emphasized with dotted lines), which serves as a built-in converter; (**b**) Variation of the photocurrent in a MAPbBr_3_ SC with metallic Gd dispersed in it showing the opening and closing of the neutron beam shutter. While the shutter is closed a white backlight is on giving an increase to the photocurrent. Measurements were performed in ambient conditions under 1 V DC bias voltage.

It is known that MAPbBr_3_ has a remarkable property that during crystallization it allows the incorporation of various objects^2^ without affecting the crystallinity. This was exploited, to embody a neutron absorbers/convertor pellet (Gd_2_O_3_) in a form of a disc in the way shown in Fig. 6a. This could simplify the detector architecture and improve the detection efficiency by increasing the useful solid angle to 4π. Contacted by graphite spray like in Fig. 1b, it demonstrates that it can detect the neutron beam (see Figs. 6b and S18), although optimization is required (thickness of the converter, form, positioning), to increase the signal to noise ratio. This will be the subject of follow-up works.

## Methods

MCNP is a Monte Carlo based general-purpose particle transport code. The MCNP model contains the geometry which in this case consists of a Gd converter foil (0.025 × 5 × 5 cm^3^), a MAPbBr_3_ SC (2 × 2 × 1 cm^3^, the actual size of the crystal used in the experiments) with graphite coating around two-third of it (0.05 cm thick) and an aluminium platform (11.505 × 20 × 4.495 cm^3^) beneath the SC. The neutron beam was assumed as a rectangular, 101.01*10^−9^ MeV monoenergetic, monodirectional beam impinging on the Gd foil. The density of the SC was set to 4 g/cm^3^ in the calculation. A mesh-based volume averaged flux tally was used to calculate the gamma flux map, while an F6 tally used to calculate the deposited energy in the crystal. The calculations were repeated with different slit openings and SC Gd foil distances. The beam flux was 10^7^ n/cm^2^/s in the calculations. MCNP was run with photon-neutron-electron mode with model physics turned on including photonuclear reactions. For every isotope the ENDF/B-VII.1 data library was used except ^157^Gd, ^79^Br and ^81^Br where Fendl3.1d database was used. For the geometry building SuperMc/MCAM software was used (http://www.fds.org.cn/en/#/, version 3.0.0)^51,52^. Gd foil in the simulations was replaced with a 5 mm thick borated polyethylene (Mirrobor^53^, 80% w/w B_4_C, the rest is polyethylene, density 1.36 g/cm^3^) to check the effect of the different converter. Calculations were repeated without converter layers too to calculate the neutron to gamma conversion inside the crystal.

The simulations with the same sized SC-were repeated for MAPbI_3_ and MAPbCl_3_ samples, the crystal geometry and density remained unchanged.

The beam of Polref contains prompt gamma photons beside the neutrons as background. A simulation was carried out to estimate the photon flux coming down the neutron guide. In this simulation the Polref instrument was simulated from the moderator with a 23 m long straight guide (the guide of Polref is bent therefore this is a conservative overestimation). The obtained prompt gamma background flux at the sample position was 1.38 × 10^4^ p/cm^2^/s. Increasing the guide opening to the size of the opening of the shutter (further overestimation) resulted in a higher gamma flux of 2.08 × 10^5^ p/cm^2^/s. These overestimated photon fluxes are far smaller than the converted fluxes even without converter layers. This is important if we would like to claim that the measured signal of the perovskite devices is coming from the converted neutrons and not from the background gamma radiation.

The crystals containing Gd_2_O_3_ pellets were tested on the ALF beamline of ISIS. The pellet diameter was 6.35 mm, containing 190 mg Gd_2_O_3_. The pellet was pressed from powder with 50 tons. 1 V DC bias was applied while the current of the crystal was measured with a Keithley 2401. Similar crystals were also fabricated with metallic Gd grains dispersed in the crystal. In Fig. 6b during the opening-open-closing periods, a blue back light was present in the room while during the closed periods the normal white light of the ALF blockhouse. The enclosure of crystal from the backlight wasn’t perfect that is why an increase during the closed shutter position is visible in the photocurrent signal. The photocurrent caused by neutrons is about 0.2–1 nA depending on the crystal size and Gd content.

## Supplementary Information

Supplementary Information 1.
