# Supplementary material for: Hybrid halide perovskite neutron detectors
Source: Sci Rep. 2021 Aug 30;11:17159. doi: 10.1038/s41598-021-95586-3 (PMC8405692; doi:10.1038/s41598-021-95586-3)
Supplement: Supplementary file 1 — Supplementary Information 1. [file 41598_2021_95586_MOESM1_ESM.docx]

Supporting Information: Hybrid Halide Perovskite Neutron Detectors

Pavao Andričević^a*^, Gábor Náfrádi^b*^, Márton Kollár^a^, Bálint Náfrádi^a^, Steven Lilley^b^, Christy Kinane^b^, Pavel Frajtag^c^, Andrzej Sienkiewicz^a,d^, Andreas Pautz^c,e^, Endre Horváth ^a^, László Forró^a^

^a^Laboratory of Physics of Complex Matter, Ecole Polytechnique Fédérale de Lausanne (EPFL), CH-1015 Lausanne, Switzerland

^b^ISIS Facility, Rutherford Appleton Laboratory, Chilton, Didcot, Oxfordshire OX11 0QX, United Kingdom

^c^Laboratory of Reactor Physics and Systems Behaviour, Ecole Polytechnique Fédérale de Lausanne (EPFL), CH-1015 Lausanne, Switzerland

^d^ADSresonances Sàrl, CH-1028 Préverenges, Switzerland

^e^Paul Scherrer Institut, Nukleare Energie und Sicherheit, PSI Villigen, 5232, Switzerland

| **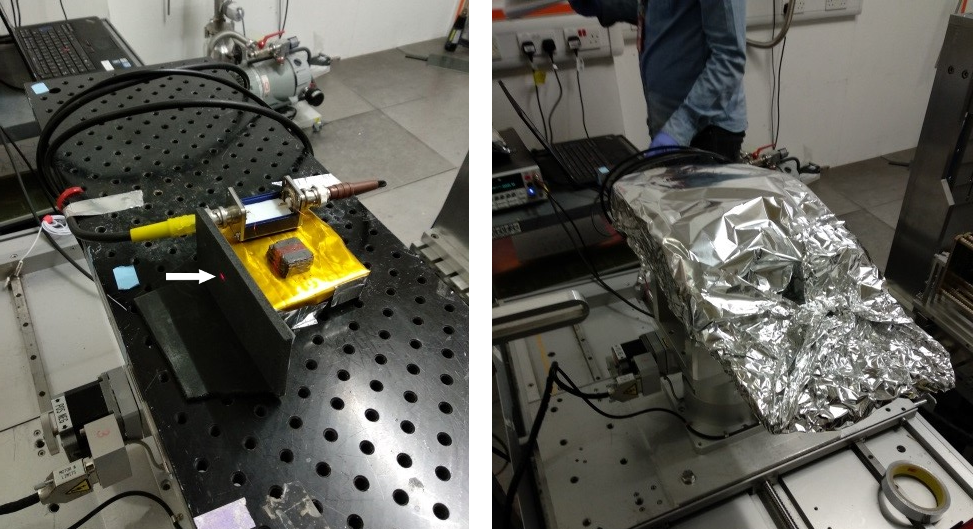**  **Figure S1.** A typical setup, borated polyethylene converter in front of a spray-coated MAPbBr_3_ crystal (left). The laser spot (white arrow) on the converter shows the centre of the neutron beam. Copper wires are connected to a sample holder connected with BNC cables to the Keithley 2400 source meter, from which the measurements were acquired. The entire setup is covered with aluminium foil to avoid visible light-related effects during the irradiation (right). |
| --- |

|  |  |
| --- | --- |
| **Figure S2.** Gamma photon generation cross-section as a function of photon energy with the materials present in the experiment, natural abundancies are used, prompt and a few delayed lines are shown too. On the right, showing only the low gamma energy part only (up to 500 keV) and not showing the common materials of the crystals. Chlorine has only two lines with a relatively small cross-section while bromine and iodine have numerous lines however with similarly small cross-sections. Gadolinium has seven low energy gamma lines with an outstandingly high cross-section while boron has only the 477 keV line. | |


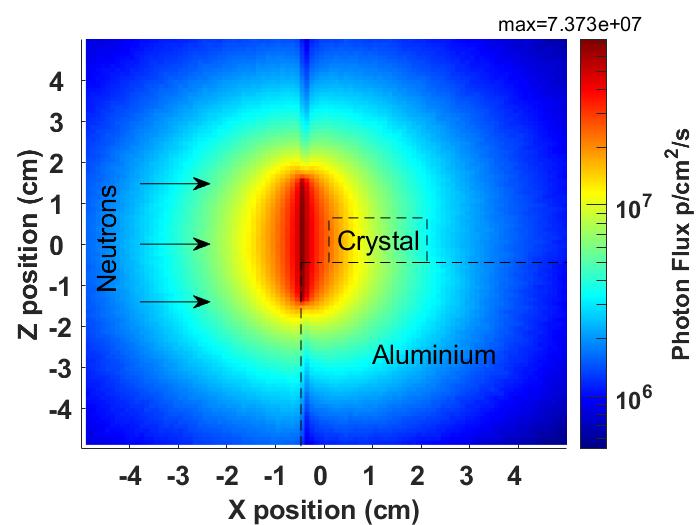


**Figure S3.** Cross-sectional view of the photon flux map surrounding the converter (Gd foil) estimated by an MCNP calculation. Neutrons are hitting the converter from the left. Gamma production is isotropic, however the self-shielding of the foil is visible at the converter plane.

| 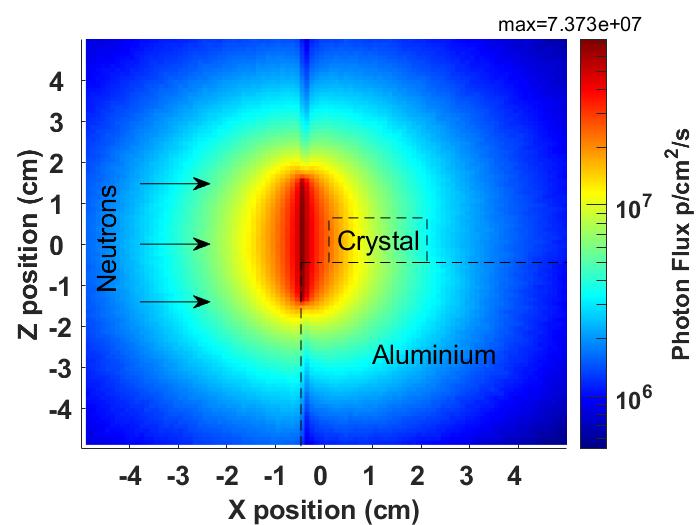 | 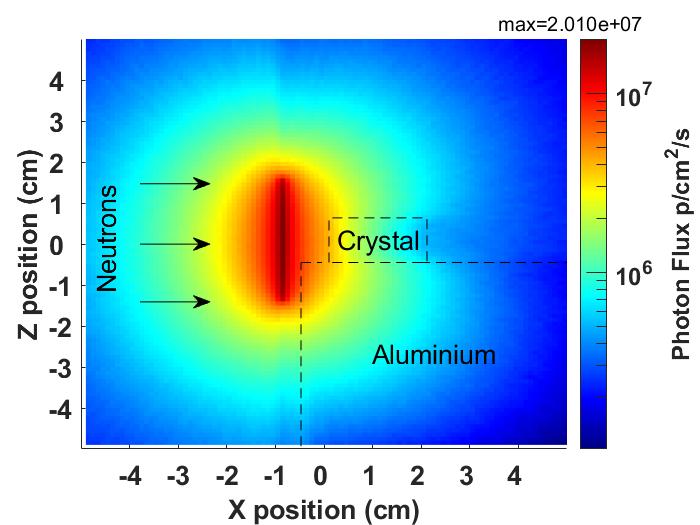 |
| --- | --- |
| **Figure S4.** On the left, there is the gadolinium simulation for comparison with borated polyethylene. The sample is MAPbBr_3_. On the right, a Mirrobor sheet (borated polyethylene, with 80% of B_4_C content, density 1.36 g/cm3, 5 mm thickness (from x=-1 cm to -0.5 cm)) replaced the Gd foil. The distance between the converter and the sample is the same in the two cases. Beam size in z is from-1.5 cm to 1.5 cm. The self-shielding is also visible for the borated polyethylene converter. | |

| 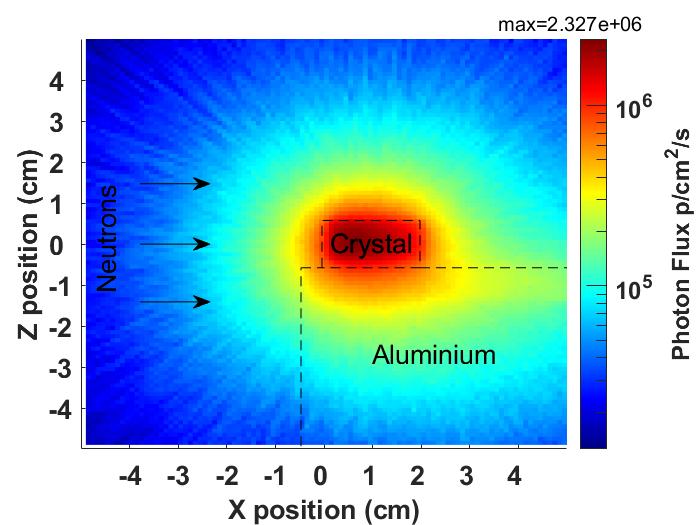 | 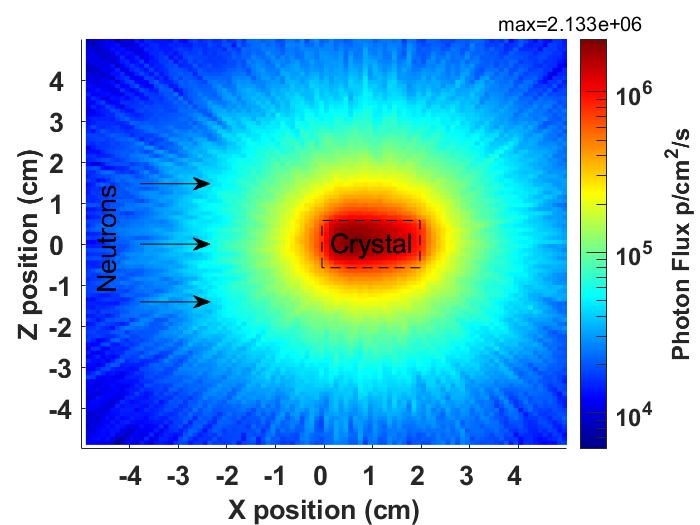 |
| --- | --- |
| **Figure S5.** Same photon flux map without any converter, the neutrons are hitting the sample (MAPbBr_3_) and the aluminium sample table underneath, the neutrons to gamma conversion happens inside the sample and in aluminium (left), the maximum is about 2.32E6 p/cm^2^/s. On the right the repeated simulation but without the presence of the aluminium table, the maximum is about 2.13E6 p/cm^2^/s. Therefore the aluminium contribution to the flux is about 0.19E6 p/cm^2^/s. | |

| 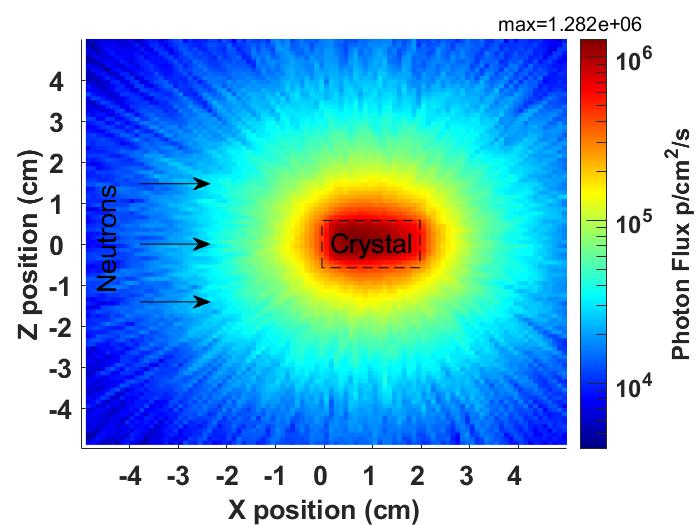 | 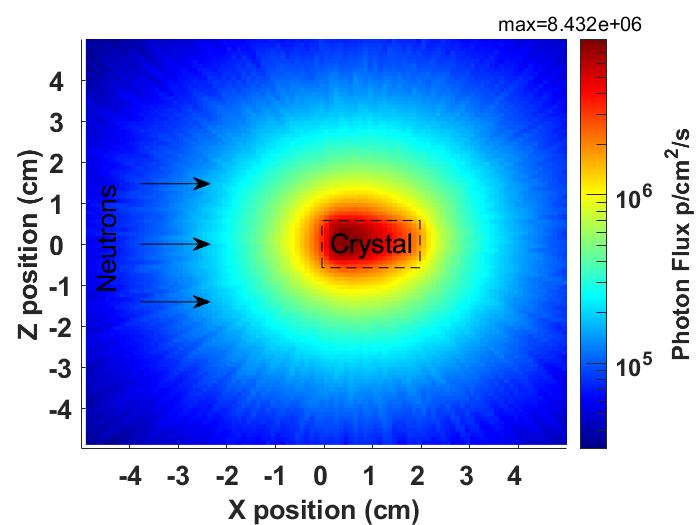 |
| --- | --- |
| **Figure S6.** MAPbI_3_ sample is on the left, same density as used for MAPbBr_3_, in vacuum, no aluminium table is used. The maximum photon flux is about 1.28E6 p/cm^2^/s. MAPbCl_3_ sample is on the right with similar arrangement, the maximum photon flux is about 8.43E6 p/cm^2^/s. | |

| 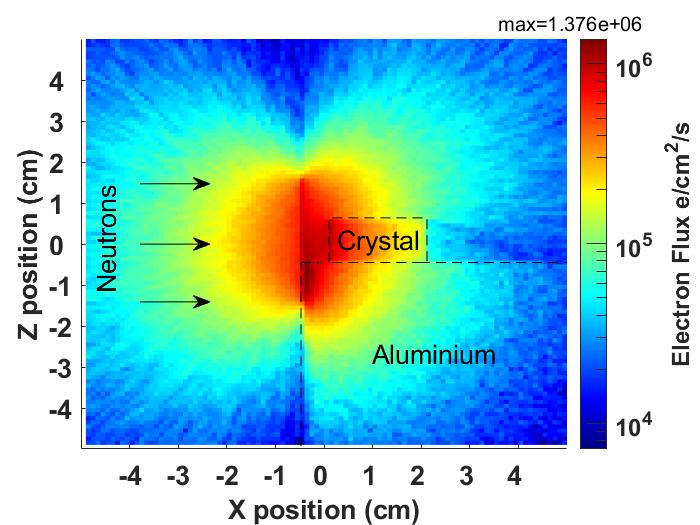 | 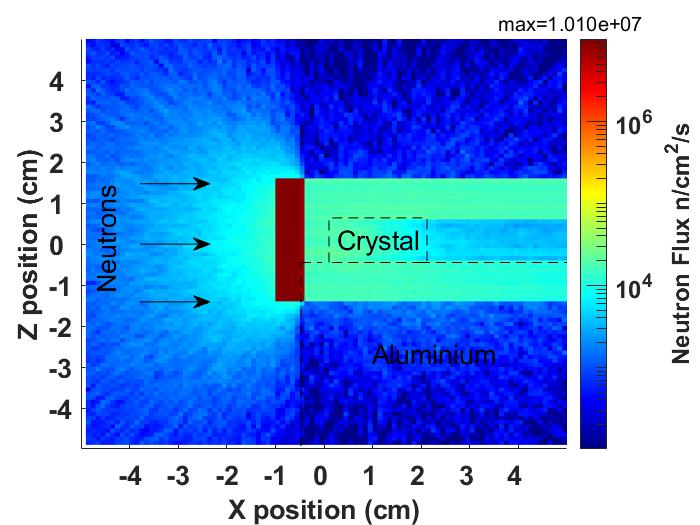 |
| --- | --- |
| **Figure S7.** Cross-sectional view of the electron flux map when the Gd converter, a MAPbBr_3_ sample and the aluminium sample table were used (left). Cross-sectional view of the neutron flux map with the same arrangement, the neutrons were started just in front of the converter (right). The colour map shows that a bit less than 0.1% of the neutrons can go through the Gd converter and reach the crystal. | |

| 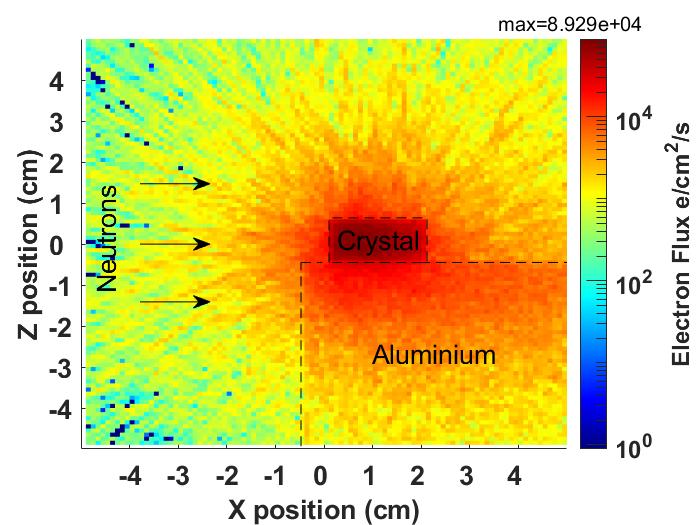 | 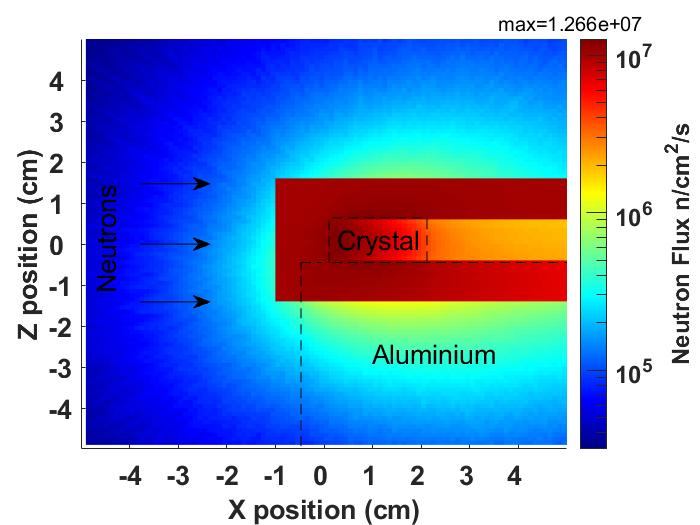 |
| --- | --- |
| **Figure S8.** Cross-sectional view of the electron flux map when a MAPbBr_3_ sample and the aluminium sample table were used (left). Cross-sectional view of the neutron flux map with the same arrangement, the neutrons were started just in front of the aluminium table (right). The neutron flux figure shows that the aluminium table is a good transparent material to be used as sample holder. While the electron flux map shows that the electrons mainly generated in the crystal and not in the aluminium. | |

| 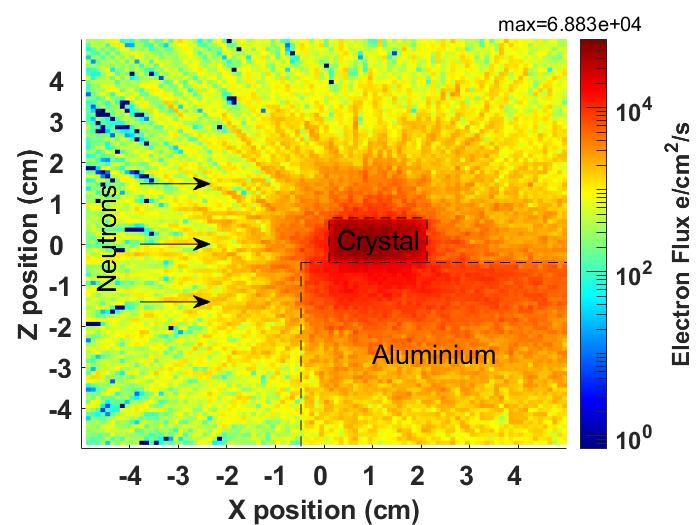 | 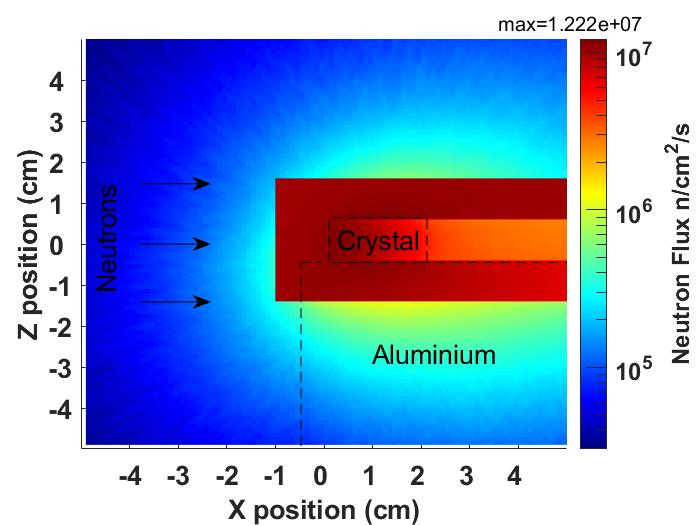 |
| --- | --- |
| **Figure S9.** Cross-sectional view of the electron flux map when a MAPbI_3_ sample and the aluminium sample table were used (left). Cross-sectional view of the neutron flux map with the same arrangement, the neutrons were started just in front of the aluminium table (right). | |

| 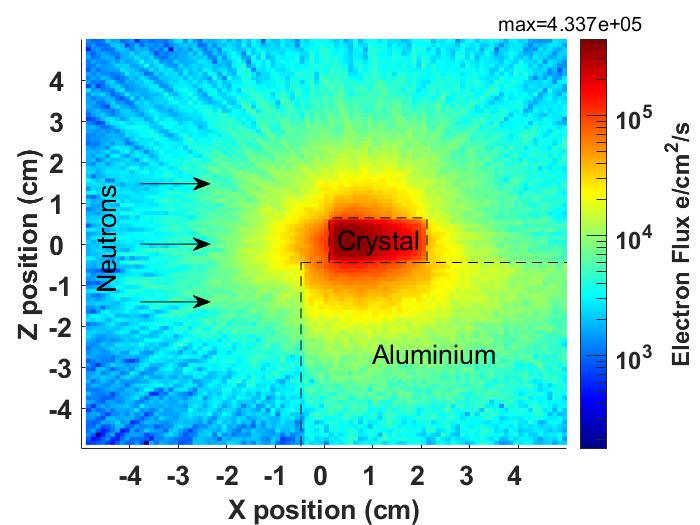 | 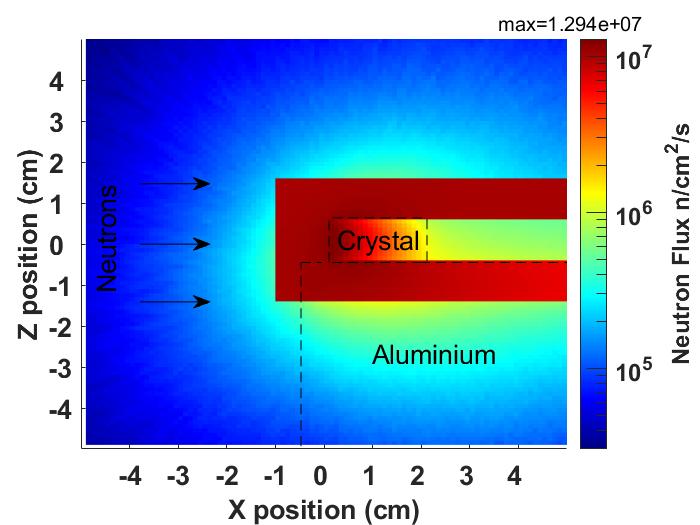 |
| --- | --- |
| **Figure S10.** Cross-sectional view of the electron flux map when a MAPbCl_3_ sample and the aluminium sample table were used (left). Cross-sectional view of the neutron flux map with the same arrangement, the neutrons were started just in front of the aluminium table (right). | |

| 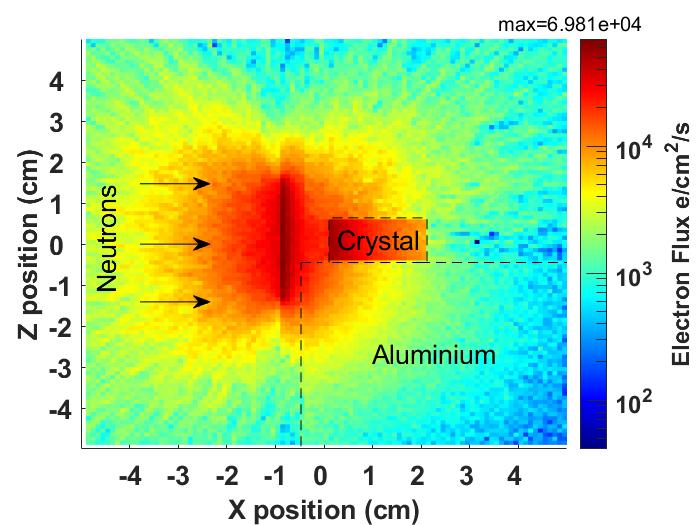 | 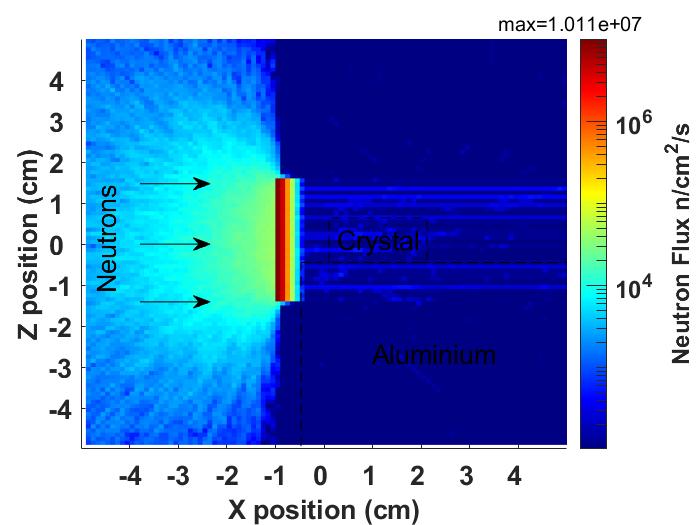 |
| --- | --- |
| **Figure S11.** Cross-sectional view of the electron flux map when borated polyethylene, a MAPbBr_3_ sample and the aluminium sample table were used (left). Cross-sectional view of the neutron flux map with the same arrangement, the neutrons were started just in front of the borated polyethylene converter (right), only a negligible portion of the neutrons could penetrate the converter layer.    **Figure S12.** Dependence of the photocurrent with the intensity of the beam. Its linear dependence can be correlated as a linear dependence of the photocurrent with the neutron flux.    **Figure S13.** Photocurrent dependence of the Gd-foil/MAPbI_3_ detector with various bias voltages. Distance of crystal to foil is 0.5 cm. Inset: Current signal in time during opening and closing of the neutron beam shutter. | |

|  |  |
| --- | --- |
| **Figure S14.** Photon spectrum in different crystals using different arrangements (left). Electron spectrum in different crystals of different arrangements (right). | |

|  |  |
| --- | --- |
| **Figure S15.** Deposited photon energy spectrum in different crystals using different arrangements (left). Deposited photon energy spectrum in different crystals due to different converter materials (right). | |

|  |  |
| --- | --- |
| **Figure S16.** Deposited electron energy spectrum in different crystals using different converters (left). The crystal material has only a minimal effect, the spectrum depends on the converter material. Deposited electron energy spectrum in different crystals without converter material (right)    **Figure S17.** Current response of the Gd-foil/MAPbBr_3_ detector at the start and finish of a 15-hour neutron irradiation. Inset: Current in time during the full irradiation cycle.    **Figure S18.** Variation of the photocurrent in a MAPbBr_3_ SC with a metallic 190 mg Gd_2_O_3_ pellet engulfed in it showing the opening and closing of the neutron beam shutter. | |

| **Table S1**: Deposited power simulated (in Watts) in samples in vacuum with 3 cm*4 cm incoming beam size, 1E7 n/cm^2^/s incoming neutron flux. | | |
| --- | --- | --- |
|  | neutron (W) | photon+electron (W) |
| MAPbBr_3_+Gd+alu table | 7.49E-14 | 7.93E-07 |
| MAPbBr_3_+borated polyethylene+alu table | 4.12E-11 | 1.42E-07 |
| MAPbBr_3_ with alu table, no converter | 1.78E-08 | 2.07E-07 |
| MAPbBr_3_ no converter | 1.68E-08 | 1.75E-07 |
| MAPbI_3_ no converter | 1.32E-08 | 1.28E-07 |
| MAPbCl_3_ no converter | 2.52E-08 | 6.75E-07 |
